# Core curriculum in pathology for future Irish medical students

**DOI:** 10.1007/s11845-021-02774-1

**Published:** 2021-09-23

**Authors:** Hilary Humphreys, Niall Stevens, Louise Burke, Mariam Sheehan, Siobhán Glavey, Mary Keogan, Erum Rasheed, the Faculty of Pathology, the Royal College of Physicians in Ireland

**Affiliations:** 1 Department of Clinical Microbiology, the Royal College of Surgeons in Ireland, Dublin, D09 YD60 Ireland; 2 Department of Microbiology, Beaumont Hospital, D09 V2NO Dublin, Ireland; 3 Department of Pathology, Cork University Hospital/University College Cork, Cork, Ireland; 4 University of Limerick School of Medicine, Limerick, Ireland; 5 Department of Pathology, the Royal College of Surgeons in Ireland, Dublin, D09 YD60 Ireland; 6 Department of Haematology, Beaumont Hospital, D09 V2NO Dublin, Ireland; 7 Department of Immunology, Beaumont Hospital, D09 V2NO Dublin, Ireland; 8 Department of Chemical Pathology, St James’s Hospital, D08 NHY1 Dublin, Ireland

**Keywords:** Chemical pathology, Clinical microbiology, Core curriculum, Haematology, Histopathology, Immunology, Medical students, Multi-disciplinary team, Pathology, Professionalism

## Abstract

Pathology is important in training to become a medical doctor but as curricula become more integrated, there is a risk that key aspects of pathology may be excluded. Following a survey of the current delivery of teaching in Ireland under the auspices of the Faculty of Pathology at the Royal College of Physicians of Ireland, suggested components of a core curriculum in pathology have been developed to be delivered at some stage during the medical course. These have been based on key principles and themes required by the Medical Council in Ireland. Professionalism is one of the core principles emphasised by the Medical Council. It includes the role of the pathologist in patient care and other professional values such as patient-centred care, clinical competencies and skills, e.g. explaining results, and knowledge under the various sub-disciplines, i.e. histopathology (including neuropathology), clinical microbiology, haematology, chemical pathology and immunology. In each of these, we suggest key aspects and activities that the medical graduate should be comfortable in carrying out. The methods of delivery of teaching and assessment across pathology disciplines have evolved and adapted to recent circumstances. Lessons have been learned and insights gained during the COVID-19 pandemic as educators have risen to the challenge of continuing to educate medical students. Integrated and multi-disciplinary teaching is recommended to reflect best the professional environment of the medical graduate who works as an integral part of a multi-disciplinary team, with the minimum dependence on the traditional lecture, where at all possible. Finally, options on assessment are discussed, e.g. multiple-choice questions, including their respective advantages and disadvantages.

**Supplementary information:**

The online version contains supplementary material available at 10.1007/s11845-021-02774-1.

## Background

Pathology is the study of disease, is fundamental to training in becoming a doctor and consists of a number of disciplines including histopathology, haematology, chemical pathology, microbiology, immunology and other related specialties [1]. As medical curricula become more integrated, and systems-based, there is concern that key aspects of pathology and laboratory medicine may be under-represented or excluded, but which are crucial to physician training [2, 3]. Recent years have seen the development of curricula in pathology in the UK and paediatrics in Ireland, to inform medical schools and medical educators on core aspects that are fundamental to the education of today and to tomorrow’s doctors [4, 5]. Therefore, it is timely for Irish pathologists to advise on core components of pathology to be delivered in medical schools, and how these core components might be delivered, now and into the future.

In Ireland, medical education is overseen, monitored and accredited, by the Medical Council. Fundamental to the guiding principles of the Medical Council is a concept of professionalism, which prioritises the patient-doctor relationship, optimises patient safety and emphasises the delivery of high-quality healthcare and diagnostics [6]. The Medical Council describes the three pillars of professionalism, as partnership, practice and performance, and within each of these are key aspects in which pathology has a fundamental role, e.g. working together as part of multi-disciplinary teams (MDTs), and promoting safety through interpreting and reporting of laboratory results, as part of accurate and reliable laboratory testing [7]. In the practice of pathology, there is considerable inter-professional collaboration and professionalism, given the close relationship between pathologists, laboratory scientists, researchers and others. Therefore, in making recommendations about the role and the input of pathology in a modern medical curriculum, these principles must underpin that concept as well as the specifics relating to pathology.

What follows are guidelines and advice on what constitutes core components of a curriculum in pathology for medical graduates, and which includes important generic themes such as professionalism. However, these are not meant to be prescriptive, except where specified, nor to mandate how the syllabus of each speciality should be delivered and by whom, as they are advisory. However, our intention is to provide some detail on topics and themes in pathology that we believe are important in medical education. While pathology teaching and learning is currently often concentrated over 2–4 semesters, much of what is outlined below may be covered elsewhere or in later years under the commonly used medical spiral curriculum, such as the pathology of tumours during rotations in oncology and electrolyte disturbances as part of endocrinology and renal medicine. Hence, it is not anticipated that all of what follows will be covered solely under specific or designated pathology teaching and learning. Furthermore, the recent COVID-19 pandemic has had a significant impact on how medical education is delivered and how assessments are conducted [8].

## Process of core curriculum development

The process of developing this core curriculum began with a survey of what was happening in Ireland at the time to determine what was being covered and by whom [3]. Subsequently, a multi-disciplinary group representing the sub-specialties was established under the auspices of the Faculty of Pathology in the Royal College of Physicians of Ireland (RCPI) to outline what would be included and to draft this document. A number of drafts were circulated and discussed amongst this group. Then the document was more widely circulated within the Faculty of Pathology, both before and after the Faculty’s Annual Meeting, which ratified the document. Feedback from this process informed the final version.

## Educational domains

Below are the broad outlines of topics and themes related to pathology that should be covered at some stage in a medical curriculum and in medical school. Some topics may be delivered later than others, e.g. haematology malignancies in the final years, rather than in the first three when basic histopathology is often delivered. These topics and themes are sub-divided into Professionalism, Clinical Competencies and Skills, and Knowledge. Under Knowledge is included what is important and what should be prioritised under the sub-disciplines of histopathology (including neuropathology), microbiology, haematology, chemical pathology and immunology. How and when these themes should be delivered within the undergraduate programme, and whether in combination, is not prescribed as local circumstances and arrangements should be considered. Examples of some possible cases and themes are included in the Appendix.

## Professionalism

### Role of the pathologist

Upon graduation, newly qualified medical doctors should be able to:

- Demonstrate an awareness of the organisational structure of pathology services in primary, secondary and tertiary healthcare settings.
- Recognise the crucial role of pathologists in healthcare delivery, diagnostics and patient care, and the importance of continuous professional development throughout their professional career.
- Identify what sub-speciality is needed and when.
- Engage appropriately with the MDT.
- Order laboratory tests to best suit the clinical need with due consideration to clinical utility, decision-making and cost.
- Use pathology reports and results correctly, and within the clinical context, with recognition of their limitations.

### Professional values

Upon graduation, newly qualified medical doctors should be able to:

- Recognise the importance of patient-centred care.
- Respect the role of other professionals in healthcare delivery and the integration of expertise with relevant colleagues.
- Recognise the importance of laboratory diagnosis in patient care.
- Recognise the evolving utilisation of molecular and genetic testing in cancer and non-cancer patient diagnostic and prognostic medicine.
- Demonstrate good practice for sample collection including phlebotomy, to avoid poor sample quality, e.g. haemolysis.
- Minimise pre-analytical errors such as avoiding mislabelling of specimens and ensuring positive patient identification.
- Recognise the importance of correct and full completion of pathology request forms, clearly indicating where required if they are clinically urgent.
- Understand the ethical considerations for some tests (e.g. when explicit consent is required), and the potential implications for the patient and their families and carers.
- Recognise the legal and ethical considerations of genetic testing and the consent process.
- Recognise their responsibility in following up on results of tests ordered with the correct interpretation of those results.
- Recognise their own limitations in the interpretation of tests, and know when and whom to ask for assistance as required.
- Accept criticism and acknowledge mistakes.
- Recognise the importance of audit and quality improvement procedures.
- Adhere to policies and follow guidelines, such as those relating to infection prevention and control, including those introduced during unforeseen circumstances such as in an epidemic/pandemic, and antimicrobial stewardship.
- Understand the rationale behind and organisation of national screening programmes and the role of pathology in these.

## Clinical competencies and skills

### Communication

Upon graduation, newly qualified medical doctors should be able to:

- Communicate effectively and appropriately with colleagues/all healthcare professionals, the MDT, patients and patient families.
- Communicate effectively with colleagues by telephone.
- Explain the meaning of test results effectively.
- Complete important documents clearly and without errors, e.g. death certificate.
- Demonstrate how pathology reports can be obtained to manage patients.

### Diagnosis and management

Upon graduation, newly qualified medical doctors should be able to:

- Understand the principles of accuracy and precision in the laboratory.
- Display an understanding of the principles of sensitivity, specificity and pre-test probability, and their role in choosing appropriate tests for a given clinical situation.
- Describe effectively the range of diagnostic investigations available for patient-centred management, including the concept of personalised medicine and targeted therapies.
- Understand the principles of pathogenesis and the role it plays in diagnosis and therapeutic management.
- Describe and have an insight into the rationale, efficient use and interpretation of each diagnostic investigation, including the need to avoid unnecessary testing that may result in false negative results, with potential adverse consequences for patients.
- Recognise the role of the MDT in reaching a diagnosis.
- Demonstrate an understanding of drug-related pathology.
- Demonstrate an understanding of molecular pathology.
- Apply the principles of antibiotic stewardship when managing bacterial infections.
- Use the principles of prescribing blood and blood products safely.
- Understand and appreciate the role of vaccines in the prevention of infectious diseases.
- Adhere to infection prevention and control measures to prevent the spread of infectious diseases.
- Apply national guidelines available for testing in acute or primary care settings.

### Data management and safety

Upon graduation, newly qualified medical doctors should be able to:

- Adhere to data management and governance guidelines, including the management of genetic data in Ireland.
- Appreciate the role of information technology systems such as BloodTrack and MedLis in ordering and reporting laboratory investigations.
- Recognise the implications of a data breach.
- Maintain patient records securely to avoid a data breach.
- Use social media platforms appropriately to avoid the inappropriate sharing of patient data.

## Knowledge

### Core histopathology (including neuropathology) knowledge

Upon graduation, newly qualified medical doctors should be able to:

- Recognise the importance of histopathology in patient diagnosis, and the crucial role of the consultant histopathologist/cytopathologist in patient management.
- Understand the range of histopathology and cytology samples, knowing which test is best to use according to the clinical situation.
- Demonstrate an understanding of a tissue sample (e.g. biopsy and fluid for cytological evaluation) pathway through a pathology laboratory.
- Demonstrate knowledge of how abnormalities of normal cellular organisation underpin the basis of disease processes (general pathology), i.e. inflammation and repair, tumour biology and behaviour, neoplasia, degenerative and haemodynamic disorders.
- Relate and apply knowledge of the aetiology and pathogenesis of diseases to the role they play in a clinical context.
- Demonstrate knowledge of the basic principles of pathophysiology necessary for understanding disease processes.
- Describe morphological changes (cellular and histological), related to mechanisms of principal pathological processes affecting organ systems, i.e. spectrum of inflammatory and repair responses, and neoplastic changes.
- Relate morphological changes to clinical manifestations in patients.
- Demonstrate basic understanding of cell signalling pathways responsible for regulating metabolism, gene expression, cell proliferation and differentiation, and explain how deregulation of these processes contributes to the development of disease.
- Recognise the mechanisms of cell death including apoptosis and necrosis.
- Demonstrate knowledge of the molecular basis of neoplasia (including molecular basis of genomic instability, DNA damage and repair, cell cycle dysregulation and epigenetic mechanisms), and cancer susceptibility with the relevant common molecular tests used in cancer.
- Recognise the role of histopathology in the classification of tumours and their grading and staging.
- Demonstrate an understanding of the mechanisms that lead to disease complications.
- Demonstrate a basic understanding of the diagnostic methodologies utilised in genomic and molecular pathology (e.g. polymerase chain reaction (PCR), next-generation sequencing and fluorescent in situ hybridisation (FISH)).
- Interpret appropriately a pathology report and recognise uncertainty if expressed.
- Demonstrate awareness of MDT meetings and the core role of histopathology in the choice of management delivered to the patient.
- Recognise the role of histochemical and immunohistochemical techniques as well as molecular testing in the diagnosis and management of disease processes.
- Understand the role of histopathology in informing cancer registries and epidemiological data.
- Demonstrate an awareness of, and the potential roles of biobanks.
- Demonstrate an awareness of the importance of post-mortem examinations in the understanding of the pathogenesis of disease, audit mechanisms in clinical practice and examination required in medico-legal deaths.
- Demonstrate an understanding of the coronial autopsy system and the requirements for death notification to the coroner.
- Understand the difference between a consented (and the principles of informed consent) and coroner-directed autopsy.
- Demonstrate an understanding of how to complete a death certificate.

### Core microbiological knowledge

Upon graduation, newly qualified medical doctors should be able to:

- Recognise the role of the clinical microbiologist in the diagnosis, management and prevention of infectious diseases.
- Recognise the signs and symptoms of sepsis, and manage sepsis appropriately.
- Describe the pathogenesis of bacterial, viral, fungal and parasitic infections.
- Associate the most likely microbial aetiology with common clinical presentations of infection.
- Identify the common risk factors that pre-dispose patients to infectious diseases.
- Recognise the classic signs and symptoms of common infections.
- Choose the appropriate microbiological specimens to send to the diagnostic laboratory.
- Communicate the findings of microbiological investigations to a MDT.
- Describe antimicrobial resistant mechanisms that have evolved in bacteria and that impact on anti-bacterial treatment.
- Understand the mechanism of action and other pharmacological properties of antimicrobial drugs.
- Adhere to general antimicrobial stewardship principles and follow national, local and institutional antimicrobial prescribing guidelines.
- Choose the appropriate empiric antimicrobial treatments to manage infections and be aware of common adverse events (e.g. side effects and drug interactions), and possible alternatives in the case of allergy.
- Rationalise antimicrobial treatments following antimicrobial susceptibility results.
- Recognise the global health problem of antimicrobial resistance and its role in preventing the emergence of new multi-drug resistant pathogens.
- Follow infection prevention and control measures to prevent the spread of infection in the clinical setting, routinely, during outbreaks and in a pandemic.
- Understand and appreciate the role of immunisation in preventing the spread of vaccine preventable infectious diseases.
- Promote public health measures and educate patients in preventing the spread of infectious diseases in the community locally and globally.

### Core haematology knowledge

Upon graduation, newly qualified medical doctors should be able to:

- Interpret haematology results within the clinical context.
- Demonstrate knowledge of laboratory tests that assist in determining the aetiology of haematological diseases and the sequence of appropriate testing from blood count, coagulation testing to bone marrow aspirate, flow cytometry cytogenetic and molecular testing.
- Describe the structure and function of the cellular components of blood.
- Recognise the biological role of haemoglobin structure and function, and inherited abnormalities of same, e.g. sickle cell disease.
- Understand iron metabolism and the interpretation of tests for transferrin and ferritin.
- Understand the significance of morphological red cell abnormalities on a peripheral blood smear and their clinical significance.
- Demonstrate knowledge of the classification, causes and clinico-pathological features of red blood cell disorders.
- Demonstrate knowledge of common disorders of platelets including thrombotic thrombocytopenic purpura and thrombocytopenia, including investigation and management.
- Recognise disorders of haemostasis and thrombosis, including common bleeding and thrombotic disorders, with an understanding of their investigation and management.
- Demonstrate knowledge of the classification, causes and clinico-pathological features of common white blood cell disorders.
- Demonstrate an understanding of the nature and types of haematological malignancies.
- Demonstrate an understanding of the clinical manifestations, evaluation and diagnosis of haematological malignancies.
- Describe in broad terms the principles of current treatment options for haematological malignancies.
- Recognise the importance of the MDT in the diagnosis and management of haematological malignancies and the role of the doctor in this process.
- Outline the multi-system consequences of haematological malignancies.
- Describe the impact of chemotherapy on the clinical, social and psychological health of the patient.
- Understand the principles of transfusion medicine, including appropriate and optimal use of blood components and a knowledge of transfusion-transmitted infections.
- Understand the process of red cell testing and the selection of patients for transfusion.
- Understand the pathophysiology, presentation and management of acute transfusion reactions.
- Demonstrate knowledge of benign red cell disorders, e.g. thalassaemia and sickle cell disease, including their pathophysiology diagnosis, management and their impact on global health.

### Core chemical pathology knowledge

Upon graduation, newly qualified medical doctors should be able to:

- Understand the role of the chemical pathologist in terms of clinical liaison for unusual or complex laboratory findings and specialist care of patients with porphyria, osteoporosis, metabolic bone disease, complex dyslipidaemia and other common metabolic diseases.
- Apply the principles on which biochemical tests are based and recognise how this is important for their diagnostic role in patient management.
- Choose appropriate tests from the range provided, know which tests to order and when, understand the particular requirements for those tests, the basics of how they may vary in paediatric or obstetric practice, and recognise situations when testing or screening is ineffective.
- Describe methodologies for common tests such as ward-based blood gas analysis, and be aware of techniques such as automated immunoassay, mass spectrometry and molecular diagnostics.
- Address how to deal with critical and potentially life-changing results, e.g. high potassium.
- Understand the concept of reference ranges and the predictive value of tests.
- Explain relevant screening programmes and their importance to patients.
- Recognise when it is not appropriate to perform screening tests in adults or children.
- Perform a bedside blood gas and glucose/ketone analysis correctly, and be familiar with safe practice and the limitations for near-patient testing.
- Describe the principles of metabolic testing, e.g. electrolytes and blood gas analysis.
- Understand the principles underlying the investigation and treatment of common fluid and electrolyte disorders.
- Investigate common toxicological problems such as paracetamol, salicylate overdose or alcohol/drug intoxication or poisoning.
- Explain the principles of therapeutic drug monitoring.
- Outline the general principles of appropriate testing for key organ-system pathologies, including renal dysfunction, liver disease, abnormal calcium, bone and mineral metabolism, myocardial infarction, congestive heart disease, endocrine disorders, hyperlipidaemia, pancreatitis, muscular and inflammatory disease, and specific protein measurements (e.g. para-protein estimation, immunoglobulins and C-reactive protein).
- Explain the scope and limitations in the use of tumour markers.
- Understand the principle of inappropriately normal results, particularly in reference to endocrine disease.
- Understand the principle of dynamic function testing, particularly in reference to endocrine disorders.
- Understand the concept of minimum retesting intervals, based on the properties of the test and the clinical situation in which it is used.
- Explain the use of biochemical tests in monitoring chronic conditions, e.g. diabetes mellitus and chronic kidney disease.

### Core immunology knowledge

Upon graduation, newly qualified medical doctors should be able to:

- Demonstrate knowledge of the range of services provided by the immunology laboratory and clinical immunology service.
- Demonstrate knowledge of the basic components and organisation of the immune system, and their physiological contribution to disease processes.
- Demonstrate an understanding of defences against infection, including natural barriers, innate and acquired immunity.
- Describe the cellular and molecular basis of the inflammatory response.
- Explain how the immune system induces immunity in the control of infectious diseases and cancer immunotherapy.
- Demonstrate an understanding of the mechanisms involved in the control of immune responses.
- Understand and describe the concept of immune dysregulation and the basic pathogenic mechanisms leading to immune-related diseases.
- Explain the principles of self-tolerance and autoimmunity.
- Demonstrate knowledge of the types of hypersensitivity reactions and their pathogenic mechanisms.
- Correlate the concepts of immunity and its changes, and relate these to the wide range of clinical manifestations seen in patients.
- Demonstrate an understanding of the basic principles of vaccine development, the different vaccine types and the rationale behind vaccination programmes.
- Identify some vaccines that have been associated with adverse vaccine reactions.
- Outline the main immune-deficiency states, how they are clinically recognised and the necessary investigations required to diagnose them.
- Explain the immunological basis for allergic reactions.
- Understand the importance of taking an allergy focussed history, and differentiating allergic disease from allergy mimics.
- Explain the assessment and key aspects of documentation of potential drug allergies.
- Outline the emergency management, and subsequent investigation where required of potential drug allergy.
- Understand the immunological basis of transfusion-related reactions.
- Demonstrate an understanding of the basic principles of transplantation.
- Demonstrate an understanding of the basic principles of immunoassays, flow cytometry and their clinical application to routine diagnostics.
- Understand terms used to describe clinical performance of assays, and relate this knowledge to how assays are used in practice.

## Delivery of teaching and learning

How teaching is delivered throughout the medical curriculum has had to be reviewed during the COVID-19 pandemic, including the recording of lectures, and with more interactive sessions online. While it is not intended to be prescriptive, some general principles are recommended. These are as follows:

- While lectures have a role, over-reliance on this method of delivery may encourage rote and superficial learning, despite their convenience, especially when the numbers available to teach are constricted, and class sizes increase. Increasingly, lectures are recorded and made available for students to subsequently access and to revise.
- Small group, interactive sessions are to be encouraged as this facilitates individual learning and comprehension. This can be in the form of case discussions with problem-orientated learning. These have been challenging to deliver during a pandemic when social distancing is mandatory but innovative approaches, including the use of IT, can compensate for the absence of face-to-face sessions.
- Multi-disciplinary case–based teaching, e.g. pathology, radiology/imaging and surgery in the case of an abdominal malignancy, highlights the importance of the MDT and provides a richer and more real-life experience for the student. This is especially true even within the disciplines of pathology itself, e.g. vaccination involving microbiology and immunology, lymphoma involving histopathology and haematology.
- While practicals are less frequently used now due to the numbers of students, the costs and for reasons of safety, every effort should be made to convey to students the role of the laboratory and how specimens are processed. This can be achieved in a variety of ways, both in practice and online/virtual, but how this is best done should be decided locally. The rotation of all students through pathology laboratories would be ideal, but this is often not feasible due to the student numbers and because many diagnostic laboratories are challenged for space. However, it may be possible for some students as part of electives or student-selected components (SSC), and if so, this should be encouraged.
- A range of healthcare professionals deliver pathology services, and where possible, these should be well represented in the faculty involved in the delivery of education in pathology. In particular, it is important that pathologists involved in the delivery of pathology services teach medical students, including recently retired pathologists, and that students fully understand their role in healthcare delivery.
- Where possible, inter-professional learning experiences should be included, such as joint teaching sessions involving students in medicine, pharmacy, science, nursing, etc. as this enriches the learning experiences, highlights their role in the MDT and provides insights into other professions and their contributions to healthcare.
- Technology-enhanced learning is increasingly important and provides the student with the opportunity to learn where and when he/she wants to. This has been especially important during the pandemic, as it assisted in maintaining social distancing. In addition, the use of, for example, e-learning can sometimes better explain more difficult concepts in the form of clinical cases, and can be used for formative assessments, and provide a mechanism for feedback [9, 10].
- Learning as part of a team and gaining an insight into the team dynamic is useful, where feasible, and could be delivered as part of project work or team presentations.

## Assessments

There are a variety of assessments that can be used and the choice of which to use will vary according to preferred medical education options, local choice and even convenience. Standard setting should where possible be carried out for key assessments.

Formative assessment, which provides the student with feedback on how he/she is doing but which does not contribute to an evaluation that decides if the student can progress (summative assessment), is to be encouraged and is usually valued by students. Such formative assessment can be in the form of multiple-choice questions (MCQs, e.g. best of five answer options as per National Board of Medical Examiners (NBME)), with feedback according to whether the student chooses the correct or incorrect option.

Summative assessment can include more than one form of assessment, which is desirable, and can be in the form of:

1. *Constructed response items: essay-type questions (ETQs)*
  These are less common now because it is accepted that such questions are difficult to set and to devise model answers. Developing marking schemes that allow for all the approaches that medical students may adopt in answering the question that can be evaluated in both a consistent and objective manner is challenging. Furthermore, correcting/assessing these is time-consuming for examiners, especially as the class size in many medical schools has increased considerably in recent years.
2. *Short notes questions (SNQs)*
  These are a variation on the ETQ but the question is more focussed and covers a narrower area for assessment. Therefore, it is easier to develop a marking template with the potential for less variation between examiners when marking such assessments.
3. *Multiple-choice questions*
  These can take the form of a stem, a question and three to five options (depends on MCQ-style being used) or extended matching questions where there is a theme, a number of stems and then five or more options as answers, where the student has to match the theme with the correct option. The stems should be context rich with a clinical vignette, and should avoid cueing to attempt to assess more than just factual recall [11]. In addition, this assessment format is used in many national licensing examinations, which are especially important for some international medical students [12]. The advantages of the MCQ are that they can be re-used, answered electronically with appropriate IT security in place and marked electronically. Hence, results are available almost immediately. Amongst the disadvantages are that many MCQs and the correct answers are passed on from one generation of medical students to another, they do not necessarily test the student’s capacity to synthesise information from a range of sources even if a case vignette is used (clinical decision-making often involves more complex processes than choosing the best option of five) and, finally, MCQs do not necessarily assess if the student can clearly articulate and coherently explain and justify a decision and a course of action.
4. *Data interpretation (DI)*
  These have the advantage that they are usually simple to devise and mark, and can be incorporated in to MCQ-type assessments. The MCQs are framed in the context of a clinical pathological scenario and the students are provided with the relevant diagnostic results, e.g. microscopic findings, culture results and biochemical tests, that they must interpret alongside the clinical vignette to answer the questions.
5. *Clinical pathological correlations (CPCs)*
  Here, the details of a case are described and the student is asked questions based on the case and he/she has to use the information provided as well as their own knowledge to answer a series of questions. Aspects of SN and DI assessments can be incorporated into the CPC by providing laboratory data for the DI and posing a question based on that and asking a general question about the underlying condition described in the case in the form of a SN. This can also involve other disciplines, jointly such as histopathology with surgery in a case of an acute abdomen due to malignancy.
6. *Oral (viva) examinations*
  These are less frequently used, given the increasingly large medical student class sizes, the challenges in ensuring that they are fair and consistent, and the advantages that they may give to students for whom English is their first language.
7. *Objective structured clinical evaluation (OSCE)*
  These are frequently used as part of assessments in medicine, surgery, child health/paediatrics, etc. They can be customised for pathology with specific questions, based on either laboratory data or other information that can be assessed in an objective and systematic way.
8. *Project work*
  This is more challenging with larger classes, and is more relevant for smaller class sizes or for SSC. Many medical schools now have SSC periods or equivalent when students spend time on projects, assignments or research. Such opportunities should be maximised for as many students as possible to gain further experience and exposure to pathology.
9. *Progress testing*
  Here, the student undertakes an assessment that is identical and repeated at regular intervals (e.g. annually or twice a year) that reflects what the newly qualified doctor should know. Hence, in the first 2 years, student scores will be predictably low but these increase as they progress throughout the course such that by the end of the course, they are achieving high scores reflecting the depth and breadth of student knowledge.
10. *Spotters*
  This is where the student must answer questions on given images relating to gross or histological changes in a clinical context.

## Supplementary information

Below is the link to the electronic supplementary material.

Supplementary file1 (DOCX 227 KB)
