# Supplementary figures and images for: Core curriculum in pathology for future Irish medical students
Source: Ir J Med Sci. 2021 Sep 23;191(4):1799–807. doi: 10.1007/s11845-021-02774-1 (PMC8458005; doi:10.1007/s11845-021-02774-1)

**Appendix**

**
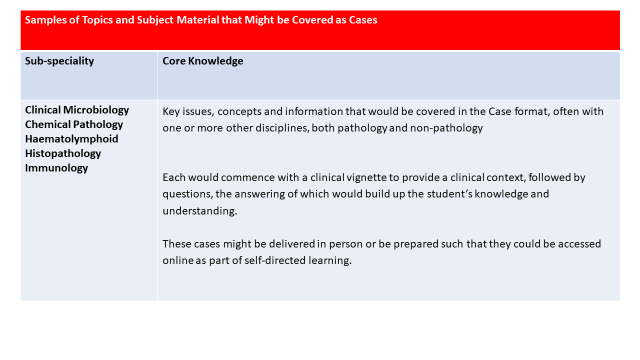
**

**
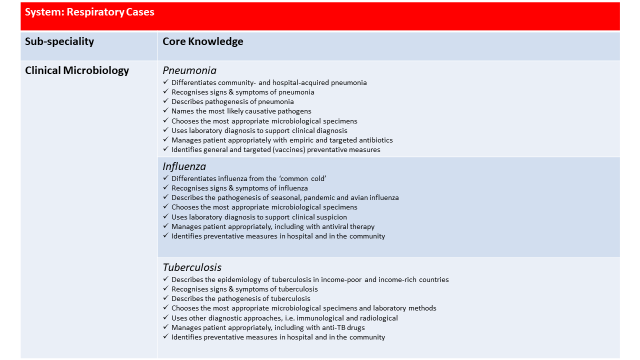
**

**
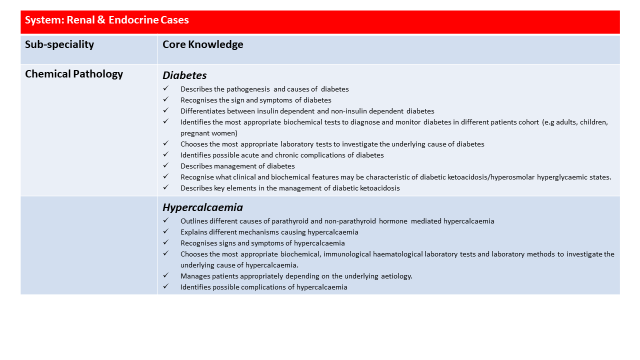
**

**
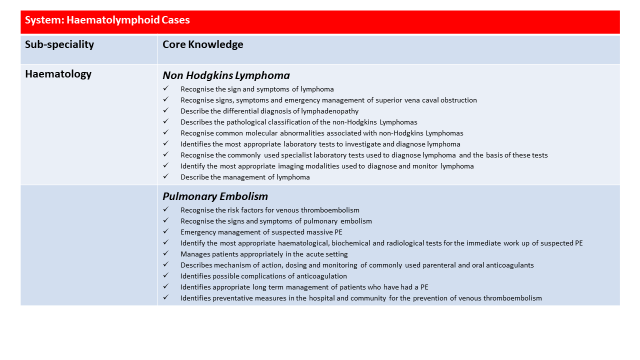
**

**
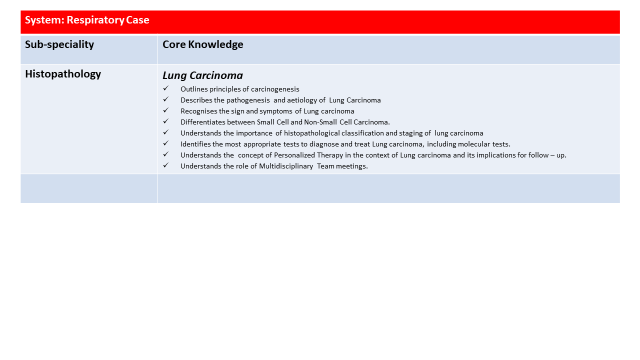
**

**
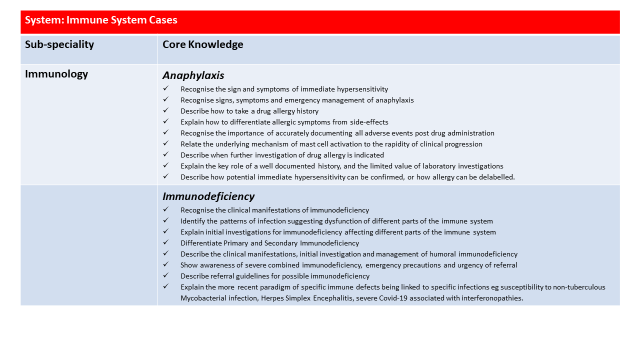
**

Supplement: Supplementary file 1 — Supplementary file1 (DOCX 227 KB) [file 11845_2021_2774_MOESM1_ESM.docx]
